# Effects of Bariatric Surgery on Depression: Role of Body Image

**DOI:** 10.1007/s11695-020-05057-3

**Published:** 2020-10-22

**Authors:** Simone C. Behrens, Konrad Lenhard, Florian Junne, Katrin Ziser, Jessica Lange, Stephan Zipfel, Katrin E. Giel, Martin Teufel, Isabelle Mack

**Affiliations:** 1 Department of Psychosomatic Medicine and Psychotherapy, Medical University Hospital Tübingen, Tübingen, Germany; 2 Department of General-, Visceral- and Transplantation Surgery, Medical University Hospital Tübingen, Tübingen, Germany; 3 Department of Psychosomatic Medicine and Psychotherapy, LVR-University Hospita, University of Duisburg-Essen, Essen, Germany

**Keywords:** Body image, Depression, Obesity, Bariatric surgery

## Abstract

**Background:**

It has been suggested that psychosocial functioning improves after bariatric surgery, but the mechanism of this effect remains unclear. We propose that body image mediates the association between %EWL and improvement in depressive symptoms.

**Materials and Methods:**

To investigate this hypothesis, we conducted a mediation analysis in longitudinal data from 52 patients after LSG.

**Results:**

%EWL had no direct effect on depressive symptoms as assessed through the patient health questionnaire (PHQ-9), but a small indirect effect that was mediated through negative evaluation of the body (BIQ-20).

**Conclusions:**

We interpret this observation in the context of complex individual etiologies of obesity and argue for a stronger focus on psychological interventions in aftercare regimes. This may be specifically relevant for patients with eating disorders or a desire for body contouring surgery.

## Introduction

Obesity is associated with significant psychosocial impairments: Compared with the normal weight population, patients have a higher rate of suffering from a psychological disorder such as depression, anxiety disorders, or eating disorders [1] and shared genetic loci for increased BMI and major psychiatric disorders are suggested [2]. Additionally, people with obesity report poor quality of life, high body image concerns, and frequent stigmatization [3–5]. While physicians typically focus on the physical and medical consequences of obesity, treatment seeking patients are often motivated by putative positive impacts on their psychosocial life [6].

It has been suggested that overall psychosocial functioning improves after bariatric surgery [4, 7], yet the likelihood of improvement and its mechanism remain unclear. A relevant mechanism of psychosocial functioning in obesity might be the interactions between disordered eating, depression and body image [4, 8, 9]. The latter is a fundamental aspect of the self-concept, because it is a constant characteristic and includes meaning of the body for self-esteem. Changing body weight does not necessarily result in a change in one’s self-concept. Therefore, body image could be relevant in obesity treatment beyond its role as an indicator of weight status reflection.

We propose that body image may influence depressive symptoms in patients that underwent laparoscopic sleeve gastrectomy (LSG). Specifically, we hypothesize that change in body image mediates the relationship between weight loss after bariatric surgery and change in depressive symptoms.

## Materials and Methods

To test the hypothesis, we analyzed data from Mack et al. [10]. The study was approved by the ethics committee of the University Hospital Tübingen (No. 727/2012BO2) and is registered at the German clinical trials register (No. DRKS00005130 available at https://www.drks.de). All procedures performed in studies involving human participants were in accordance with the ethical standards of the institutional and/or national research committee and with the 1964 Helsinki declaration and its later amendments or comparable ethical standards.

### Participants and Procedure

Seventy-five patients (66% of eligible patients) who had undergone LSG at the University Hospital Tübingen participated in the study. All participants were assessed before (pre) and 21–80 months after surgery (post). Further details on the recruitment and data assessment procedure are reported in Mack et al. [10].

For the present analysis, only participants with complete pre and post data in depression (PHQ-9), %EWL, and at least one measure of body image (BIQ-20) were considered (*n* = 52). About 44.2% of participants were male. Participants were mean 44.3 (SD 11.5) years old, and 36.5% of the sample had a clinical diagnosis of depression when they underwent surgery. At follow-up the mean time since surgery was 48.5 (SD 12.9) months, and time since surgery was a significant but in terms of effect size negligible predictor of %EWL (*R*^2^ = 0.08; *F*(1,50) = 4.61*, p* < .05).

### Materials

The investigated variables were assessed as follows:

- *Percentage of excess weight lost (%EWL)* was determined as percentage of excess weight (referring to a body mass index of 25 kg/m^2^) that was lost since the LSG. In seven (13%) of the included patients only self-reported weight was available at follow-up.
- *Body image* was assessed using the Body Image Questionnaire (BIQ-20; [11, 12], English version in Lamade et al., [13]). The BIQ-20 comprises of 20 items and assesses body image on the two subscales: “negative evaluation of the body” (NEB) and “perception of body dynamics” (PBD). NEB assesses a negative attitude towards the own body and a perceived lack of sense of coherence with the own body. PBD, on the other hand, assesses feelings of power and health as well as interest in bodily activities such as sexuality or dancing. While high scores on NEB indicate a lack of well-being with the own body, PBD is inversely scored and high values reflect a positive body image. Conceptually, the BIQ-20 matches the research question of the present project, since it focuses not on evaluations of specific body parts but rather on the body image as part of the self-concept. The BIQ-20 has proved valid and change sensitive in various studies [14–16].
- *Depression* was assessed using the depression section of the Patient Health Questionnaire (PHQ-9; [17]). The PHQ-9 assesses depressive symptoms according to ICD-10 criteria over the last 2 weeks with nine items. It closely matches internationally renowned concepts of depression and is sensitive in monitoring severity changes in clinical samples [17].

### Statistical Analysis

Statistical analyses were performed in IBM SPSS Statistics version 26. Bivariate associations between the model variables were analyzed with Pearson correlations. Mediation analysis was performed according to the approach by Hayes [18] utilizing the PROCESS macro for SPSS version 3.4. Inference about the indirect effect was determined by bootstrapping, reporting 95% bootstrap confidence intervals. The number of bootstrap samples was set at 10.000. Effects of the mediation analysis are reported as unstandardized effects (*b*). Since our analyses were based on change scores, we explored in post-hoc analyses how the mediation models changed when the analysis was restricted to the patients with strong effects in depressive symptoms and body image. Three subsamples were generated using median splits of the sample based on PHQ-9 and BIQ-20 change scores.

## Results

### Sample Characteristics

From pre to post LSG, mean BMI significantly decreased from 47.8 (SD = 8.5) to 36.8 (SD = 8) kg/m^2^, *t*(51) = 12.14, *p* < .001, *d* = −.94, resulting in a mean %TWL of 23.3% (SD = 12.3) and %EWL of 50.6% (SD = 27.8). About 83% of the sample yielded an %EWL of at least 25%; 52% achieved an %EWL of at least 50%. PHQ-9 scores significantly decreased from 9.3 (SD = 6.1) to 6.9 (SD = 6), *t*(51) = 2.83, *p* < .01, *d* = −.28; BIQ-20 NEB significantly decreased from 38.8 (SD = 7.5) to 27.3 (SD = 10.5), *t*(51) = 9.2, *p* < .001, *d* = − .89; and BIQ-20 PBD improved from 24.3 (6.8) to 27.4 (SD = 9.2), *t*(48) = − 2.73, *p* < .01, *d* = .27.

### Correlations

Despite of the overall significant weight loss and reduction in depressive symptoms, %EWL was not correlated to the change in PHQ-9 (*r* = .04, n.s.) or BIQ-20 PBD (*r* = .26, n.s.), but only with BIQ-20 NEB (*r* = − .48). Similarly, %TWL was not correlated to change in PHQ-9 (*r* = .03, n.s.) but to BIQ-20 PBD (*r* = .32) and to BIQ-20 NEB (*r* = −.48). However, PHQ-9 change was moderately associated with changes in BIQ-20 NEB (*r* = .31) and BIQ-20 PBD (*r* = −.38).

### Mediation Analysis

Consistent with the observed correlations, the total effects model for %EWL predicting PHQ-9 change was not significant (*F*(1,50) = 0.74, *p* < .79, *R*^2^ < .01). However, the mediation model that included BIQ-20 NEB change as a mediator was overall significant with 14% of the variance in PHQ-9 change explained (*F*(2,49) = 4.05, *p* < .05, *R*^2^ = .14). The indirect effect that depicts the influence of %EWL on PHQ-9 change mediated by BIQ-20 NEB change was small, but significant (*b* = − 0.04, (95% BCa CI), (− .08; − .01)), suggesting that higher %EWL leads to reduced depressive symptoms, mediated through reduced negative evaluation of the body.

**Fig. 1:**
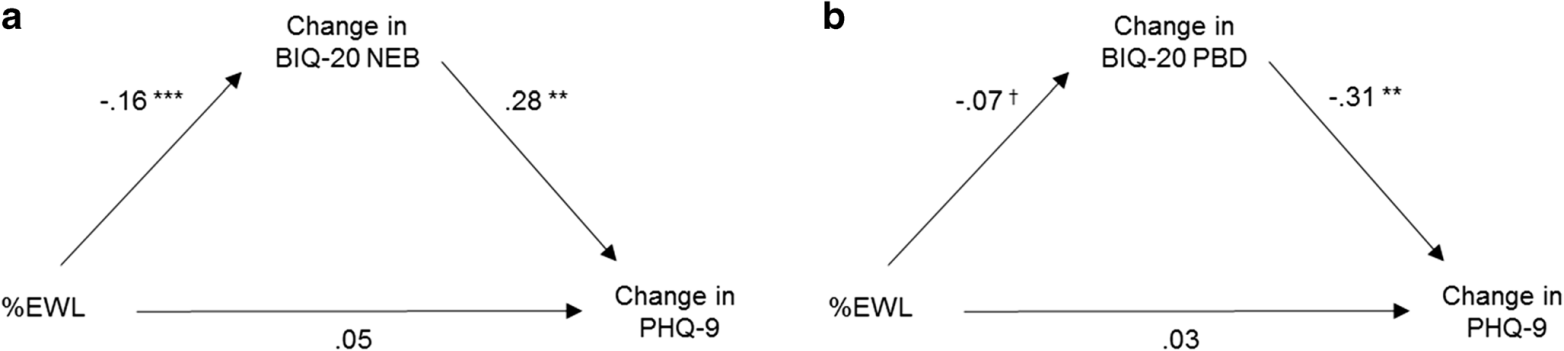
Mediation model investigating the relationship between weight loss and improvement in depressive symptoms after bariatric surgery. Displayed effects are direct effects. *** *p* < .001, **p < .01 ^†^*p* < .10.%; EWL percentage of excess weight lost after surgery, BIQ-20 Body Image Questionnaire [12], NEB negative evaluation of the body subscale, PBD perception of body dynamics subscale, PHQ-9 patient health questionnaire depression module [17]. **a** Analysis with BIQ-20 NEB as a mediator (*n* = 52). **b** Analysis with BIQ-20 PBD as a mediator (*n* = 49)

The mediation analysis for BIQ-20 PBD (conducted with *N* = 49) yielded less clear results. Again, the total effects model for %EWL predicting PHQ-9 change was not significant (*F*(1,48) = 0.02, *p* < .88, *R*^2^ < .001). The mediation model that included BIQ-20 PBD change as a mediator was overall significant with 16% of the variance in PHQ-9 change explained (*F*(1.48) = 4.25, *p* < .05, *R*^2^ = .16). However, the indirect effect that depicts the influence of %EWL on PHQ-9 change mediated by BIQ-20 PBD change was not significant (*b* = −0.02 (95% BCa CI) (− .05; .003)]. Analogue effects were observed if %TWL was used as a predictor instead of %EWL. Figure 1 illustrates the mediation analysis.

Post hoc analysis explored how the mediation models change when only subgroups of patients with high benefit in depression or body image were considered. Therefore, median splits of the sample based on PHQ-9 and BIQ-20 change scores were conducted. The median split resulted in the following subsets: patients with a PHQ-9 improvement of at least − 2 points (*N* = 30), BIQ-20 NEB improvement of at least − 11 points (*N* = 28), and BIQ-20 PBD improvement of at least 3 points (*N* = 26). The restriction of the sample size turned the total effect models to overall statistically not significant. Interestingly, the focus on patients with change in depressive symptoms and on patients with change in BIQ-20 PBD reduced the proportion of explained variance in the mediation model to *R*^2^ < .08. However, the focus on patients with change in BIQ-20 NEB increased the proportion of explained variance to *R*^2^ = .22.

## Discussion

This study analyzed for the first time the influence of body image on depression after LSG. So far, recommendations for management before and after surgery largely focus on maximizing and maintaining weight loss and metabolic outcomes [19]. Our results suggest that the frequently observed improvements in psychosocial functioning after bariatric surgery are not directly due to the resulting weight loss. Rather, bariatric surgery appears to trigger complex behavioral, physiological and cognitive changes involving body image that in sum improve both health and psychosocial outcomes.

We observed that although depressive symptoms moderately improved following bariatric surgery, this improvement was not correlated to the percentage of excessive weight that was lost. In our mediation analysis, we only observed a small yet significant *indirect* effect of %EWL on depressive symptoms that was mediated through a change in BIQ-20 NEB (but not PBD). Post hoc analyses suggested that the observed effects were due to a subgroup of patients which improved in NEB following bariatric surgery and in which body image was significantly linked to depressive symptoms. Still, a large proportion of variance in improvement of depressive symptoms remained unexplained, suggesting that it is a complex process that cannot be reduced to one or two determinants.

The present study adds on to recent work [9] suggesting that body image is a key mechanism for bariatric surgery outcomes. So far, a common interpretation of associations between disordered eating, body image, and depression was that obese people have “healthy” body dissatisfaction due to their excess weight that normalizes when they adopt healthy eating habits and normalize their weight. Instead, our study along with other literature [8, 9, 20, 21] suggests that a subgroup of patients with obesity presents with pathological body image in the sense of body dissatisfaction, overvaluation of weight and shape, and low self-esteem. It is known that these symptoms can even deteriorate after bariatric surgery, since most patients suffer from excess skin [22]. Body image disturbances are also core symptoms of eating disorders, with high explanatory power for their maintenance and outcome [16, 23, 24]. Consequently, for this subgroup, psychoeducational group aftercare may be indicated and has already been shown to be most effective in patients with high psychological burden [25].

It must be noted that we ran all statistical analyses in this study based on change scores, which may have caused bottom effects. Although we tried to tackle this problem in our post-hoc analyses, our sample size was too small to run an analysis with clinically depressed patients only. Also, there is broad consensus that depressive symptoms typically have a complex origin, and so are the mechanisms that may bring relief. While we see our assumption confirmed that body image is relevant for change in depressive symptoms, our results also imply that body image is only one among multiple factors that influence depressive symptoms. Due to sparse data assessment, theoretically plausible contributions of life time depressive disorders or the need for revision or contouring surgery have not been investigated.

Overall, the present study contributes to an improved understanding of bariatric surgery outcomes. Our observations suggest that in a subgroup of LSG patients with poor psychological outcome, body image is a mediating factor of depression. Current preparation and aftercare regimes for patients that undergo bariatric surgery often focus on nutrition and physical activity, but hardly target psychological components [26, 27]. Based on our observations, we suggest incorporating interventions to improve body image into aftercare regimes. This may be specifically relevant for patients with eating disorders or a desire for additional body contouring surgery. Further, we propose that future studies should target individual perspectives on obesity causes and consequences in the evaluation of treatment outcome.
